# Storm surge variation along the coast of the Bohai Sea

**DOI:** 10.1038/s41598-018-29712-z

**Published:** 2018-07-27

**Authors:** Jianlong Feng, Delei Li, Yan Li, Qiulin Liu, Aimei Wang

**Affiliations:** 1 National Marine Data & Information Service, Tianjin, China; 2 Key Laboratory of Ocean Circulation and Waves, Institute of Oceanology, Chinese Academy of Sciences, Qingdao, China; 3 Function Laboratory for Ocean Dynamics and Climate, Qingdao National Laboratory for Marine Science and Technology, Qingdao, China; 4 Key Laboratory of Research on Marine Hazards Forecasting, Beijing, China; 5 Center for Ocean Mega-Science, Chinese Academy of Sciences, Qingdao, China

## Abstract

The present study mainly investigates the storm surge variations at different temporal scales using hourly tide gauge data in the Bohai Sea. The seasonal variation, inter-decadal variation, long-term trend and the tide-surge interaction were analyzed separately. The results show that the storm surges in the southwest Bohai Sea are larger than those in the north. The storm surges were more serious in winter (Oct. to Mar.) than in summer half of the year. Significant inter-decadal variations exist in the Bohai Sea, and the extreme storm surge events have been intensifying since 2010. Storm surge intensities at three of the tide gauges (Qinhuangdao, Huludao and Tanggu) exhibited a decreasing trend from 1980 to 2016, with trends significant at the 95% level at Qinhuangdao and Tanggu. Significant tide-surge interactions were observed at all four tide gauges. The tide-surge interaction that results in peak surges mostly occurs during the flood and ebb tides. There is a statistically significant negative correlation between storm surge intensity and the Arctic Oscillation (AO) at Longkou and Tanggu, while there is a significantly positive correlation between storm surge intensity and the Siberian High (SH) at Huludao, Qinhuangdao and Tanggu. A linear regression analysis revealed that the variations of the AO and SH explained 19–48% of the variations in the storm surge intensity in the Bohai Sea.

## Introduction

**Figure 1:**
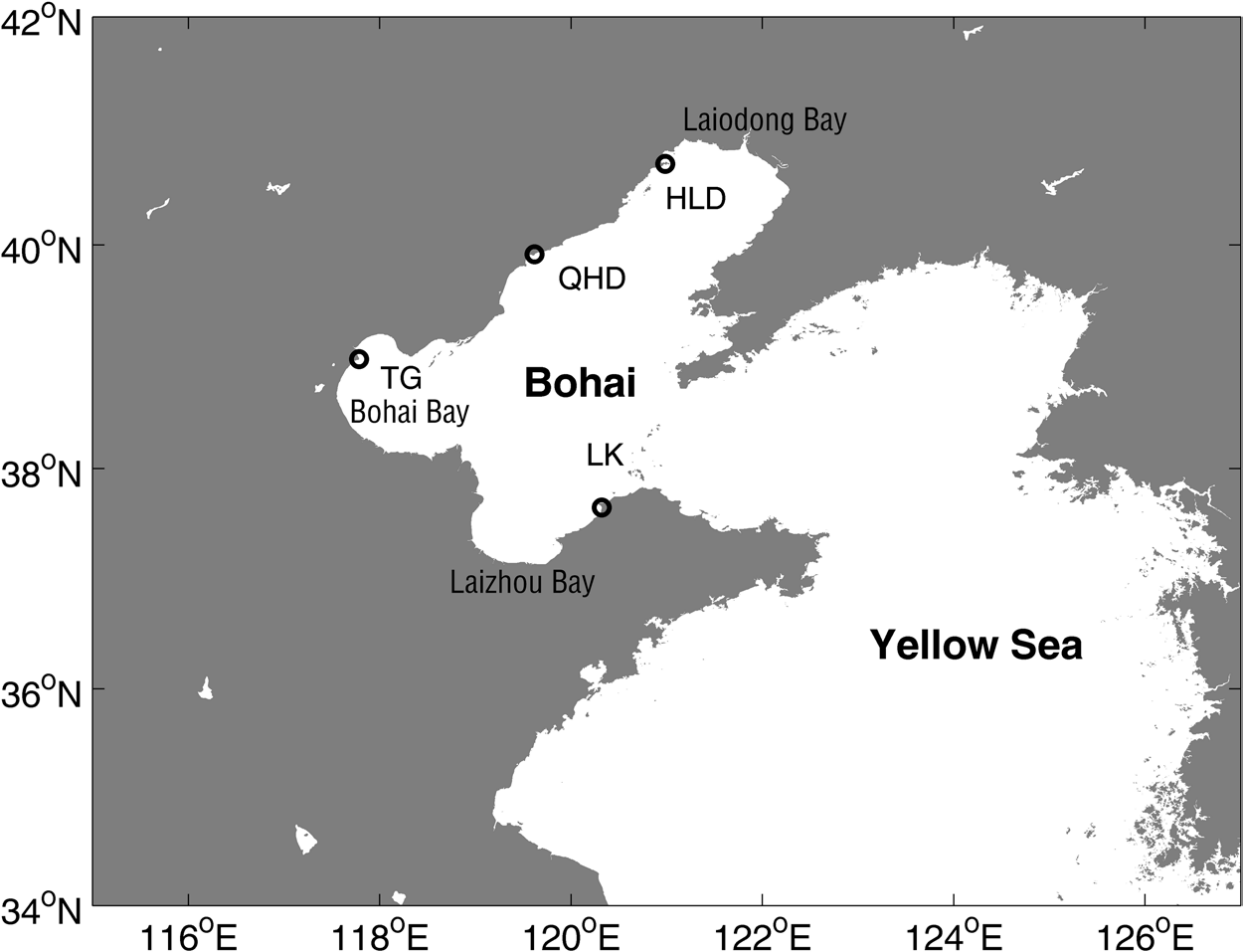
The locations of the Bohai Sea and the four tide gauges (Huludao (HLD), Qinhuangdao (QHD), Tanggu (TG) and Longkou (LK)).

Because of the rich and fertile land, feasible port access, fishing access and abundant transport connections, millions of people are crowded around coastal zones. As one of the major geophysical risks of coastal areas, storm surge is often associated with heavy casualties and property losses^1–3^. The storm surge disasters in China caused economic losses of 10.5 billion Chinese Yuan, 148 deaths and affected 11.5 million people annually from 1990 to 2010 (China Marine Disasters Bulletin). As the only semi-enclosed inland sea in China, the Bohai Sea has an area of approximately 78,000 km^2^, and its proximity to Beijing, the capital of China, makes it one of the busiest seaways in the world (Fig. 1). Although the Bohai Sea is less susceptible to typhoons, with only approximately 11 typhoons that have passed the Bohai Sea since 1980 (http://tcdata.typhoon.gov.cn), cold-air outbreaks and extratropical cyclones often bring serious storm surge disasters, especially in the winter^4,5^. The rapid economic development and population increases have accelerated the storm surge disaster risks in the coastal regions along the Bohai coast^6^.

The growing concern about climate change has motivated numerous studies to evaluate the effects of climate change on the mean and extreme sea levels^7–13^. Many of these studies have indicated that the storm surge has also undergone through remarkable changes during the past several decades, and these changes were important parts of the extreme sea level changes in some areas. Bromirski *et al*.^14^ found that the winter storm surge in San Francisco showed a significant increasing trend beginning in 1858. Ullmann *et al*.^15^ found that the maximum annual sea level had risen twice as fast as the mean sea level because of the changes in the storm surges. Weisse *et al*.^13^ found that the storm surge in the North Sea from 1912 to 2010 featured an increasing trend. Wahl and Chambers^16^ believed that the changes in extreme sea level along the southeast coast of North America were affected by the storm surge caused by hurricanes. Unlike the changes in the mean sea levels, the changes in storm surges throughout the world have clearly shown spatial differences over the past several years.

Recently, risk assessments have been widely utilized to plan for coastal flood mitigation under climate change scenarios^17–21^. However, most studies assumed that the statistical characteristics of the storm surges remain unchanged in the long run^22,23^. Some assessments also considered the variations in storm surges, most of which were represented by the characteristics of changes in tropical cyclones^6,24–26^. A better understanding of the characteristics and trends of storm surges is important for flood risk assessments, coastal planning as well as for defining an indicator of the potential coastal climate changes.

The storm surges in the Bohai Sea are mostly caused by cold-air outbreaks and extratropical cyclones^27–29^, which are strongly impacted by other large-scale systems such as the Arctic Oscillation (AO), Siberian High (SH), East Asian Winter Monsoon and East Asian jet stream^30–32^. The storm surges in the Bohai Sea have been studied using numerical models since the 1980s^33–36^. Some researchers focused on case studies or verification of storm surges hindcast^34,37,38^, some studied the effects of the cold-air outbreak paths on the occurrence of storm surges^4,39^, while others concentrated on land reclamation patterns and changes to the coastline due to storm surge^35,40^. Few studies have investigated the long-term changes in storm surges in the Bohai Sea. Feng *et al*.^5^ analyzed the characteristics of storm surges in the Bohai Sea from 1961 to 2006 using a numerical model. Zhang and Sheng^41^ simulated the extreme sea levels in the northwest Pacific Ocean, and they obtained the 50-year return levels in these areas. However, these works were all carried out using numerical models, and they lacked comprehensive verifications by comparing the modeled results with observed storm surges in the Bohai Sea.

Using long-term sea level observations from 4 tide gauges, we comprehensibly investigated the storm surge variations over the Bohai Sea spatially and temporally, and the relationships between the storm surges and climate indices were also analyzed. The outline of this paper is as follows: section 2 provides a brief description of the datasets used in this study, section 3 presents the results of the analysis and discussions of the characteristics of the storm surges in the Bohai Sea, and section 4 presents the main conclusions drawn from this study.

## Datasets

Hourly sea level data from 4 tide gauges, Huludao (HLD), Qinhuangdao (QHD), Tanggu (TG) and Longkou (LK), along the Bohai coastline were used in this paper (Fig. 1). The datasets were obtained from the marine monitoring stations in China from January 1980 to December 2016. The data have been carefully quality controlled, including the data spikes and spurious data^42^. At the LK station, the data were available for less than 60% of the year in 1990, and these data were excluded from analysis. The storm surge data were obtained using the harmonic analysis method^43^.

The AO index, which is characterized by pressure anomalies of one sign in the Arctic with the opposite anomalies centered at 37–45°N, was obtained from the University of Washington (http://jisao.washington.edu/ao/). The Siberian High (SH), which is the accumulation of cold (very cold) dry air in northeastern Eurasia, was calculated using the data from the National Center for Atmospheric Research (NCAR). The SH was calculated using the method established by Gong and Wang^44^, in which the index I was used to represent the intensity of the SH:

1 $$I=\frac{\sum _{n=1}^{N}{P}_{n}{\delta }_{n}\,\cos \,{{\rm{\Psi }}}_{n}}{\sum _{n=1}^{N}{\delta }_{n}\,\cos \,{{\rm{\Psi }}}_{n}}$$

where P_n_ is the sea level pressure at point n, and Ψ_n_ is the latitude of n. When P_n_ ≥ 1028 hPa, δ_n_ = 1; otherwise, δ_n_ = 0. The selected area is located at 30°N~70°N and 60°E~120°E.

## Results and Discussion

### Seasonal variability

**Figure 2:**
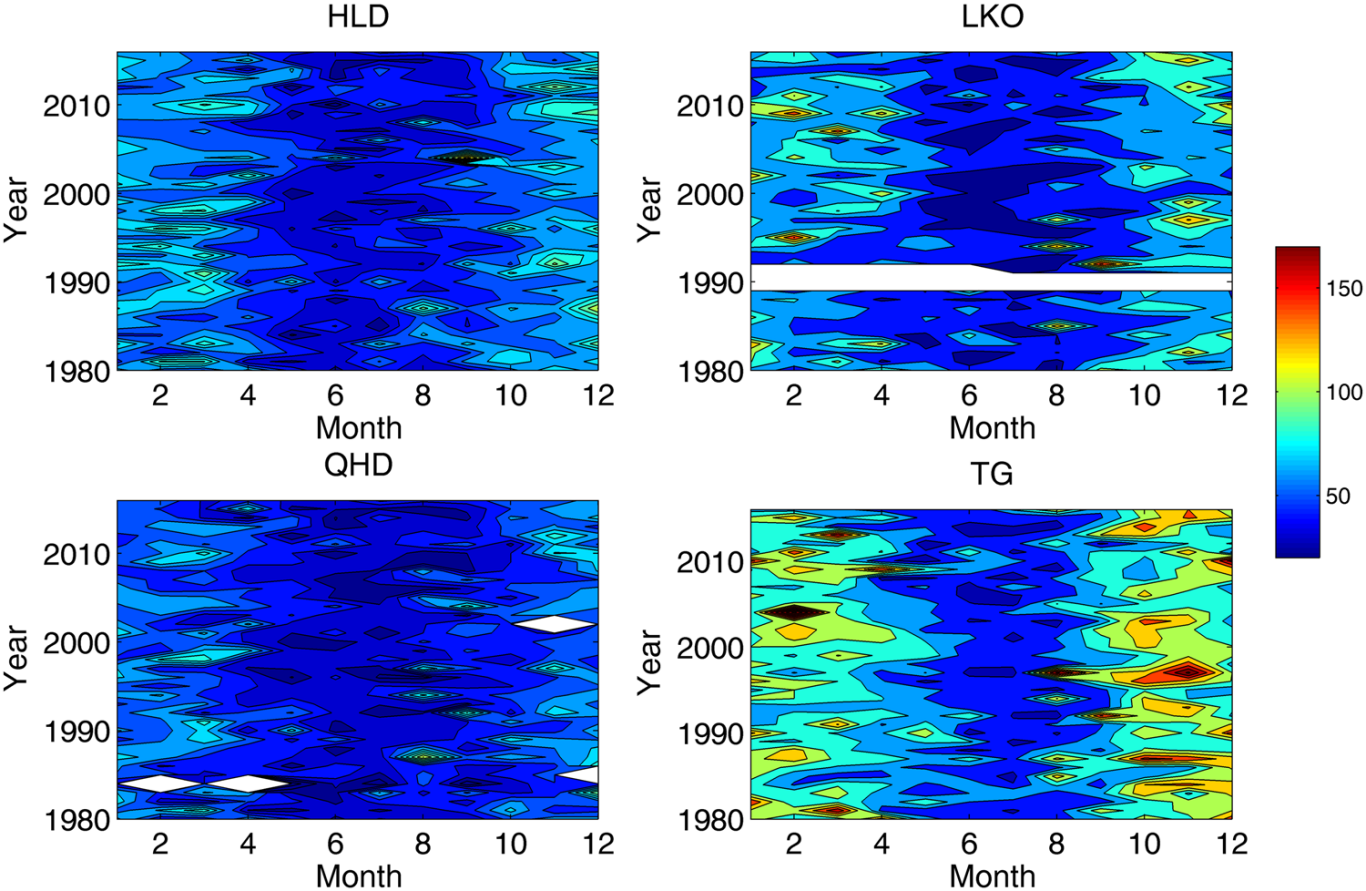
Monthly maximum storm surge levels (in cm) at the four tide gauges, white areas indicate missing values.

The monthly maximum storm surge levels at 4 tide gauges were calculated to describe the annual storm surge cycle along the Bohai coastline. The 37-year results are shown in Fig. 2. It can be found that the monthly maximum values exhibit clear seasonal variability at all 4 of the tide gauges. The storm surge levels were larger in winter half year (October to March) than those in summer half year, which is generally consistent with the intra-annual variability of cold surge frequencies in the Bohai area^45^. The storm surge level at TG was the largest among all tide gauges, with the largest value in February 2004 (250 cm) and the smallest value in May 2012 (25 cm). The largest storm surge at LK occurred in February 1995 (162 cm), and the smallest value occurred in June 2010 (16 cm). The storm surge levels at HLD and QHD were not as large as those at the other two tide gauges. The largest value at HLD occurred in September 2004 (126 cm), while the smallest value occurred in June 2010 (13 cm). The largest value at QHD occurred in August 1987 (111 cm), and the smallest value occurred in May 1984 (4 cm). It can be seen that the storm surges along the southwest coast of the Bohai Sea were larger than those along the northern coast. It should be noted that some high storm surge levels were generated in July and August of some years, which is thought to be caused by typhoons passing the Bohai Sea. No clear change was found in the seasonal variability at the four tide gauges during the past 37 years.

The seasonal and spatial characteristics of the storm surge in the Bohai Sea can be well explained by the characteristics of cold-air outbreaks and extratropical cyclones, which are the two main causes of the storm surges in the Bohai Sea. Ding and Krishnamurti^46^ and Zhu *et al*.^47^ revealed that cold-air outbreaks, which are led by the cold-core high from Siberia and Mongolia, mostly occur during the late autumn and early spring. The spatial characteristics of the storm surge in the Bohai Sea were strongly correlated to the cold-air outbreak tracks, as shown in Zhao and Jiang^4^ and Mo *et al*.^39^. All four tracks, from the northwest (44%), west (33%), north (18%) and east (9%), caused high easterly winds over the Bohai Sea, resulting in more serious storm surges in Bohai Bay and Laizhou Bay than in the other regions of the Bohai Sea.

### Tide-surge interaction

Previous studies^48–51^ showed that significant nonlinear tide-surge interaction exists between the tide and surge at some locations, especially for shallow seas and estuaries. The tide-surge interaction can significantly affect the amplitude and timing of storm surges; thus, the surge peaks mostly occur during the rising or falling tide.

**Figure 3:**
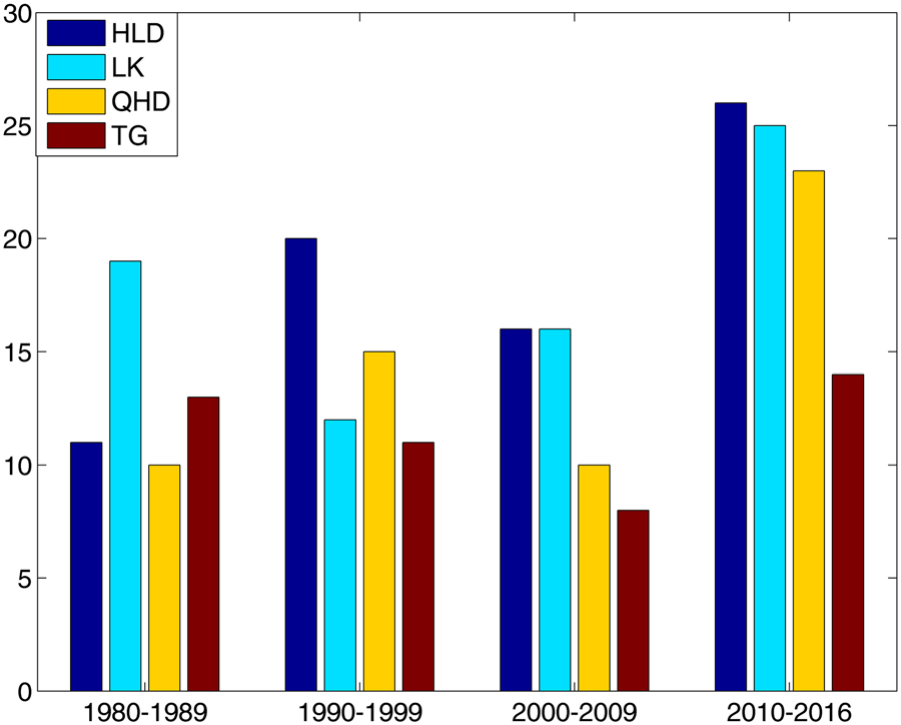
Distribution of the surge peaks above the 99.9% threshold with respect to the high tides at 4 tide gauges (Huludao, Longkou, Qinhuangdao and Tanggu), the colors represent the different surge levels, the y-axis is the number of the storm surges, and the x-axis is the timing before (negative) and after (positive) the high tide.

Using the method established by Haigh *et al*.^12^, the role of the tide-surge interaction in the distribution of surges was analyzed in this work. According to the tide cycles at the tide gauges, the tides were divided into thirteen (or twenty-five) hourly bands. Then, the timings of the surge peaks relative to the nearest high tide were calculated. If a tide-surge interaction exists at the tide gauge, the number of surges (exceeding the threshold *u*, which is the 99.9^th^ percentile of the water level) in each band will differ from one to another. The distributions of the observed surge peaks above the 99.9% levels with respect to the timing of high tides at the 4 tide gauges are shown in Fig. 3.

The results showed that a tide-surge interaction existed at all four tide gauges. There were surge peaks during both the rising and falling tide periods. The features of the tide-surge interaction varied among the different tide gauges. At HLD, the surge peaks mostly occurred during the falling tide period, which was approximately 1–4 hours after the high tide. At LK, the surge peaks were more frequent during the falling tide, which was approximately 2–5 hours after the high tide. At QHD, there were surge peaks during both the falling and rising tide periods, which were 5 hours before and 2 hours after the high tide, respectively. At TG, the surge peaks were more centralized than those at the other three gauges, with the most frequent peaks occurring 4 hours after the high tide. The results also showed that no clear distribution rule existed between the different surge levels. It should be noted that few storm surges occurred exactly during high tide at all four tide gauges; in particular, no surge peak occurred during high tide at LK.

### Storm surge variations

**Figure 4:**
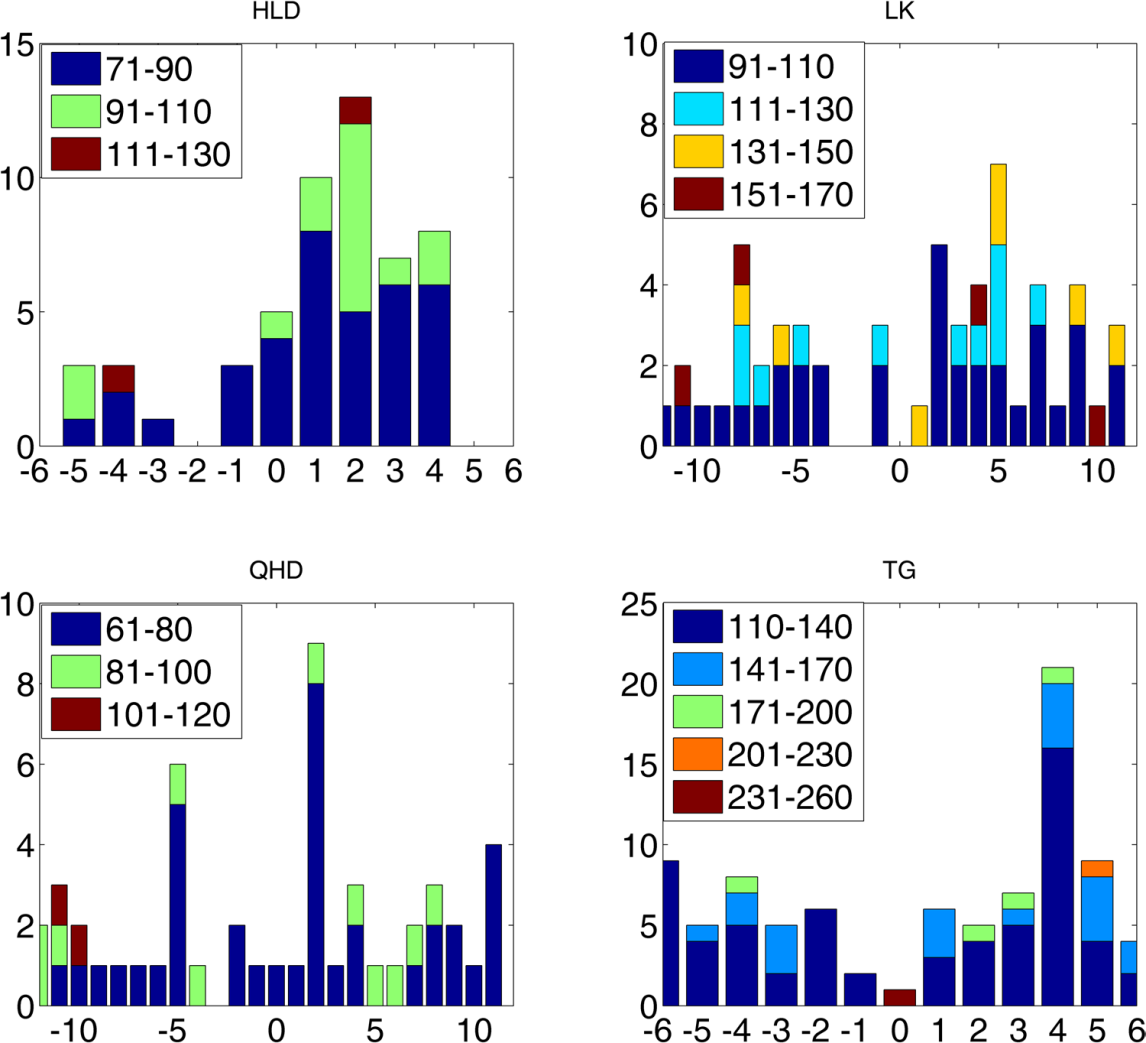
Number of storm surges (above the 99.9% level) during each ten-year band at HLD, LK, QHD and TG.

The number of the storm surges (above the 99.9% level of storm surges) in each ten-year band was calculated to study the decadal variations in the extreme events of storm surge at each tide gauge. To obtain an independent storm surge event, at least 96 hours were required between events. The results (Fig. 4) show that the decadal variations in storm surge at the four tide gauges share some common features, with the most surge events during 2010 and 2016 at all four tide gauges, although there were only 7 years in this band. This result indicates that the frequency of the storm surges intensified from 2010–2016. There are also pronounced differences among the four tide gauges in terms of decadal variations in storm surge. The fewest storm surges occurred from 1980 to 1989 at HLD, from 1990 to 1999 at LK, and from 2000 to 2009 at TG, while the numbers were small from both 1980 to 1989 and from 2000 to 2009 at QHD. The results also show that the total number of surges is smallest at TG, which means that the larger surge levels were more concentrated at TG than the other tide gauges.

To study the long-term trends of the storm surges, the method described by Zhang *et al*.^52^ was used in this work. The storm surges were represented by three indices:

1. Storm surge count: annual number of storm surges above a given threshold
2. Storm surge duration: the annual number of hours for which the storm surges were above a given threshold
3. Storm surge intensity: the annual total integral of the surge level curve above a given threshold

**Figure 5:**
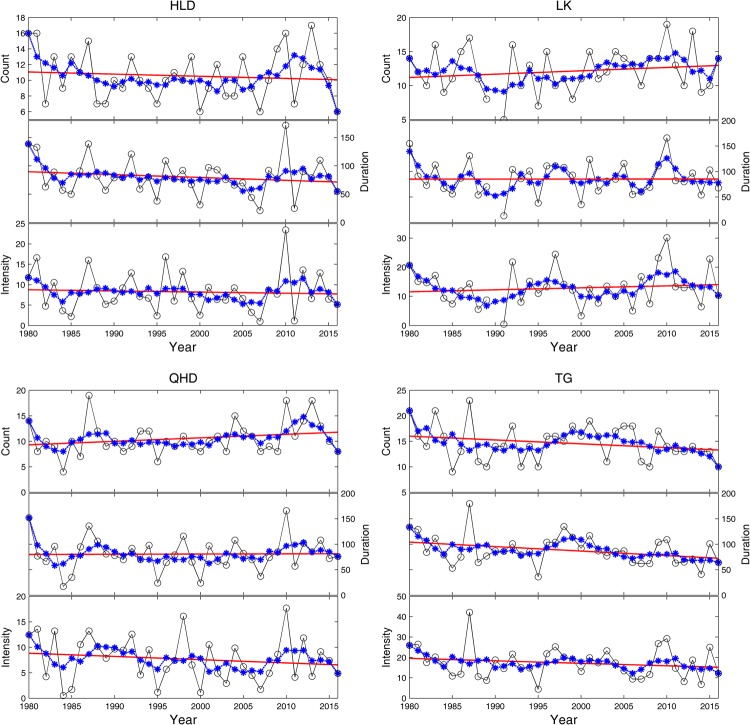
Annual storm surge count (black dotted line) (up), annual storm surge duration (black dotted line) (middle), and annual storm surge intensity (black dotted line) (bottom) at HLD, LK, QHD and TG. Red lines indicate the linear trend (red line), and the blue lines represent the 5-year running mean.

In this work, the 99^th^ percentile of the storm surge level was used as the threshold. The same temporal interval (96 hours) was required for detecting independent storm surge events. The annual variations of the storm surge indices are given in Fig. 5 at the four tide gauges. Table 1 lists their long-term trends.

**Table 1:** Long-term trend of the storm surge count, duration and intensity at 4 tide gauges.

Tide gauge	Count	Duration	Intensity
HLD	−0.028	**−0.506**	−0.028
LK	**0.049**	0.006	0.068
QHD	0.069	0.040	**−0.064**
TG	**−0.076**	**−0.884**	**−0.122**

^a^Trends with significant at the 95% confidence level are in bold.

Figure 5 shows that storm surges are the most frequent and strongest at TG when using the 99^th^ percentile of water levels as the storm surge threshold, which is different from the results shown in Fig. 4, which use the 99.9^th^ percentile as the surge threshold. The results also show that the interannual variability of the indices at all tide gauges was quite large, whereas the interdecadal variability was relatively weak. At HLD, the storm surge count ranges from 6 to 17, with the largest value in 2013 and the smallest values in 2000, 2007 and 2016. The storm surge duration ranges from 21 hours to 172 hours, the storm surge intensity ranges from 1.01 hour·m to 23.45 hour·m. The largest value of the two indices occurred in 2010, and the smallest value of the two indices occurred in 2007. At LK, the storm surge count ranges from 5 to 17, with the largest value in 1987 and the smallest value in 1991. The storm surge duration ranges from 13 hours to 166 hours, the storm surge intensity ranges from 0.47 hour·m to 30.15 hour·m. The largest value of the two indices occurred in 2010, and the smallest value of the two indices occurred in 1991. At QHD, the storm surge count ranges from 4 to 19, with the largest value in 1987 and the smallest value in 1984. The storm surge duration ranges from 17 hours to 166 hours, and the storm surge intensity ranges from 0.51 hour·m to 17.69 hour·m. The largest value of the two indices occurred in 2010, and the smallest value of the two indices occurred in 1984. At TG, the storm surge count ranges from 4 to 19, the largest value occurred in 1987, and the smallest value occurred in 1984. The storm surge duration ranges from 36 hours to 180 hours, the storm surge intensity ranges from 4.44 hour·m to 42.12 hour·m. The largest value of the two indices occurred in 1987, and the smallest value of the two indices occurred in 1995.

At HLD, the storm surge count, duration and intensity exhibit similar characteristics, with high values around the 1980s and 2010s, and negative linear trends exist in all three indices. The linear trend of the duration was significant at the 95% level. At LK, the storm surge indices feature quite similar variation patterns, with large values from 2007 to 2013 and low values from 1985 to 1994. Positive linear trends were obtained for all 3 indices, and the linear trend of the storm surge count was significant at the 95% confidence level. At QHD, the three indices were relatively high from 1985 to 1994 and 2008 to 2012. The storm surge count and duration showed positive linear trends, while a significant negative linear trend was found in the intensity. At TG, the storm surge count and duration showed similar characteristics. These two indices were relatively high from 1995 to 2005. Negative linear trends were obtained for the 3 indices, and all of them were significant at the 95% confidence level. At all of the four tide gauges, the storm surge indices were high around 1980. The storm surge intensity at all stations except LK showed decreasing trends, and the trends were significant at the 95% confidence level at QHD and TG.

### Large-scale conditioning of storm surge genesis

Woodworth and Blackman^10^ indicated that it is important to determine whether the variability of extreme storm surges is related to climate variations when analyzing the changes in storminess. Many studies have indicated that the changes in the storm surge were related to the regional climate variations, such as the North Atlantic Oscillation (NAO), Arctic Oscillation (AO), and El Niño-Southern Oscillation (ENSO)^5,10,53,54^. The storm surges in the Bohai Sea were mainly caused by cold-air outbreaks and extratropical cyclones^27–29^. Studies show that one possible reason for the changes in cold-air outbreaks is the recent change in the air circulation system, particularly the change in the behaviors of the winter monsoon system across eastern Asia^55–60^. The changes in the East Asian winter monsoon (EAWM) can affect the Siberian High through the upper wave train and the lower cold temperature^55,61,62^. However, the link between winter monsoons and temperature is quite complex, and the link is dependent on not only the strength of the cold-air outbreak but also the tracking of multi-modal changes. Park *et al*.^63^ and Li *et al*.^64^ pointed out that in different AO phases, the tracks of the cold surges as well as the persistent period and damage degree in two different AO phases differ, revealing that the changes in the cold-air outbreaks and extratropical cyclones are highly related to the Arctic Oscillation (AO) and Siberian High (SH)^44,65–68^. The changes in the AO and SH can affect the intensity and frequency changes of the cold-air outbreaks and extratropical cyclones in North China. When the AO is positive, the surface pressure in the polar region is low, which will lock the cold Arctic air in the polar area. In contrast, high pressure in the polar region can help frigid polar air move to middle latitudes^69^. Gong and Ho^57^ found that the warmer winters throughout inland extratropical Asia were driven by the weakening of the SH. Wang *et al*.^70^ found that the tracks of the cold-air outbreak were affected by the AO and SH. The tracks from the west are mainly impacted by the negative phase changes of the AO, the northwest tracks are typically engendered by cold air masses at high latitudes that move with the East Asian winter monsoon, and the tracks from the north are largely affected by the warm high anomaly from the Ural Mountains. Meanwhile, the Siberian High under northwest tracks is weaker than that under west and north tracks. The relationship between the AO and SH and the storm surges in the Bohai Sea was analyzed to gain insights into the dynamical mechanism of the storm surge changes in this area.

**Figure 6:**
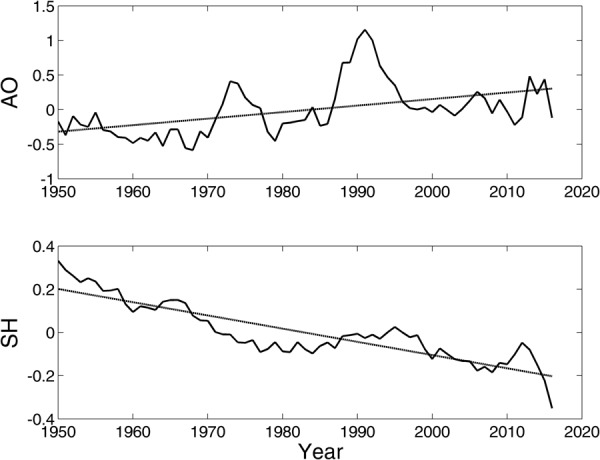
The 5-year running mean (black line) and linear trend (black dotted line) of the AO and SH from 1950 to 2016.

The 5-year running mean and linear trends of the AO and SH (annual mean from September to May) are shown in Fig. 6. The results showed that clear decadal variations and positive linear trends existed in the AO index from 1950 to 2016. The increase rate is approximately 0.01 per year. Meanwhile, a decreasing trend existed in the SH from 1950 to 2016. The rate of decrease was approximately −0.006 per year. Previous studies^66,67,71^ showed that the synoptic-scale baroclinic waves will decrease when the AO increases. As a result, the cold waves that affect North China will decrease. Wang and Ding^72^ indicated that the intensity of the cold-air outbreak will decrease if the SH decreases. The changes in the AO and SH may explain the interdecadal variations and long-term trends obtained in section 3.3. The cold waves that affected the Bohai Sea decreased during 1950 and 2016 because of the changes in the AO and SH. As a result, the intensity of the storm surge in the Bohai Sea also decreased during this period (results at HLD, QHD and TG). It should be noted that positive linear trends of the storm indices were obtained at LK. These differences may be caused by the tracks of the cold-air outbreaks. The cold-air outbreaks along the northwest track vary from north to northeast, the cold-air outbreaks from the west track usually bring strong northwest winds, the cold-air outbreak from the north track commonly induce strong northeast winds, and the cold-air outbreak from the east track mainly result in high easterly winds. The winds of these cold-air outbreaks blow the water in the Bohai Sea to the west. Because the LK tide gauge is located in the southeast of the Bohai Sea, the long-term changes of the storm surges at this tide gauge is different from those at the other three tide gauges. When the storm surges at the other three tide gauges decrease, the storm surge at the LK shows a positive trend.

The correlations between the storm surge intensity over the Bohai Sea and climate indices, such as the AO and SH, were calculated. The results show that the storm surge intensity was correlated with the AO and SH (Table 2). The storm surge intensities at LK and TG were significantly negatively correlated with the AO index. No significant correlations were found at HLD and QHD. At the same time, significant positive correlations were found between the storm surge intensity and SH at HLD, QHD and TG. Moreover, the correlation coefficients after detrending were also calculated. The results show that the correlation between the storm surge intensity and SH became non-significant after detrending at only TG. The interdecadal variation of the AO and SH may explain the interdecadal variation of the storm surge in the Bohai Sea.

**Table 2:** Correlation between the storm surge intensity and SH, AO at HLD, LK, QHD and TG, the correlation after detrending (in the brackets), the coefficient of determination (R2) in the linear regression analysis method.

	HLD	LK	QHD	TG
AO	−0.08 (−0.09)	**−0.49 (−0.49)**	0.16 (0.14)	**−0.41** (**−0.51)**
SH	**0.42 (0.39)**	−0.05 (0.04)	**0.43 (0.31)**	**0.38** (0.17)
R^2^	0.23	0.20	0.19	0.48

**Figure 7:**
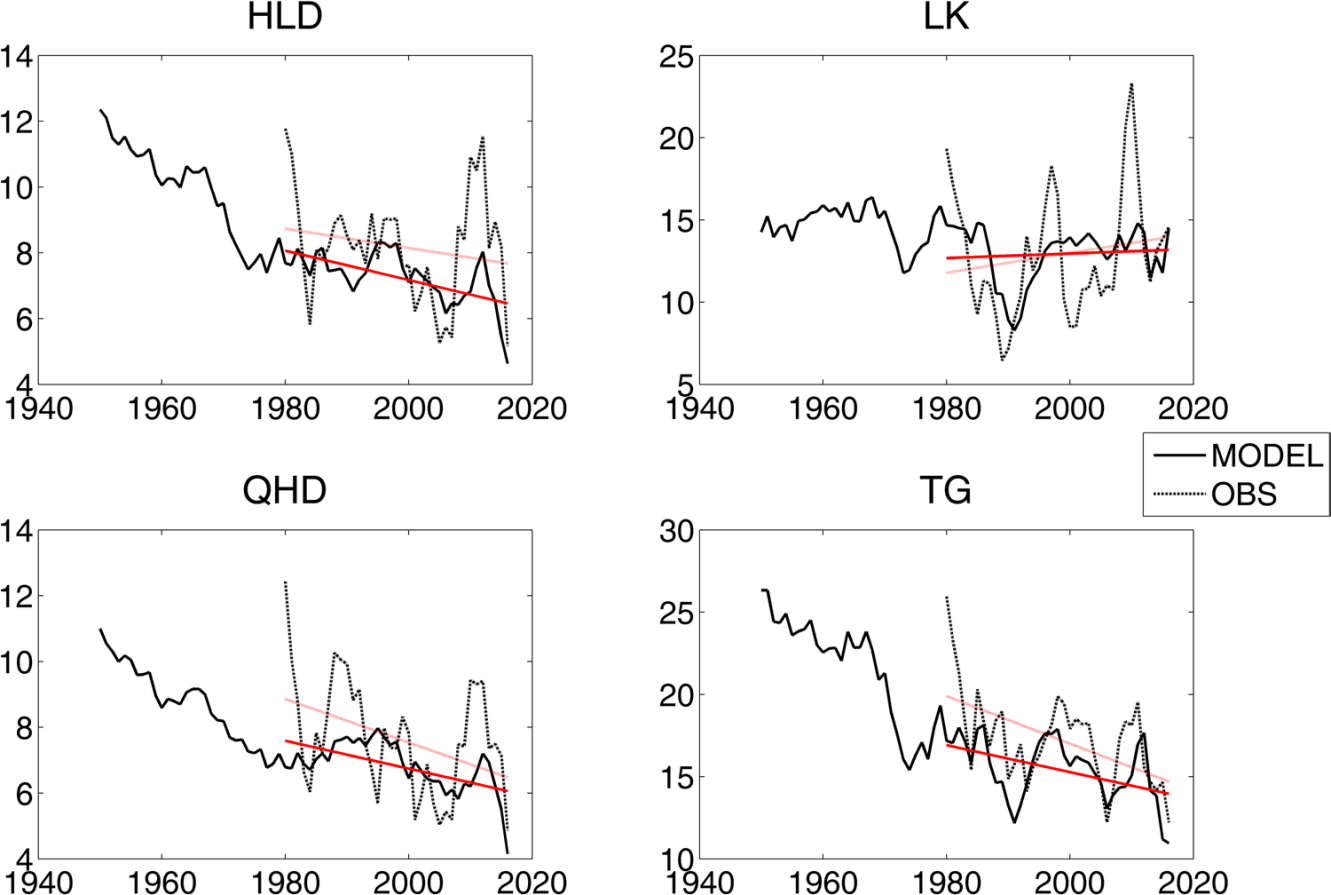
Linear regression analysis results of the storm surge intensity at HLD, LK, QHD and TG, the results from the regression model (solid line), the results of the observations (dotted line), and corresponding linear trends from 1980 to 2016 (red line).

Thus, a linear regression model can be used to construct the statistical relationship between the storm surge intensity and AO and SH,

2 $${S}_{t}(x)={A}_{a}(x)A{O}_{t}+{A}_{s}(x)S{H}_{t}+{\xi }(x)$$

where ${S}_{t}(x)$ is the storm surge intensity in year *t* at location *x*. The regression coefficients are the first order proportional to the correlations ${C}_{a}(x)$ and ${C}_{s}(x)$,

3 $${A}_{a}(x)={C}_{a}(x){S}_{a}/{S}_{ss(x)},\,{A}_{s}(x)={C}_{s}(x){S}_{s}/{S}_{ss}(x)$$

where ${S}_{a}$, ${S}_{s}$ and ${S}_{ss(x)}$ are the standard deviations of the AO, SH and storm surge intensity at the selected location. The results of the linear regression analysis of the storm surge intensity at the 4 selected tide gauges are shown in Fig. 7. The coefficients of determination (R2) are listed in Table 2. The results show that the linear regression model results are similar to the observations at all four tide gauges, especially the long-term trend. To some extent, the results of the linear regression analysis can also reproduce the interdecadal variations. However, it seems that the results from the linear regression model were gentler than the observations at all 4 tide gauges. The R2 values show that the changes in the AO and SH can explain 19–48% of the changes in the storm surge intensity at the 4 tide gauges. Thus, the storm surge intensity from 1950 to 2016 was calculated using a linear regression model. The results showed that the storm surge intensity displayed a clear decreasing trend in the period from 1950 to 2016 at all 4 tide gauges.

## Conclusions

The growing concerns about climate change have motivated numerous researchers to study the effects of climate change in coastal areas. As one of the major geophysical risks in coastal areas, the changes in storm surge related to climate change have drawn more concern in recent years. In this work, hourly sea level data from four tide gauges, which are Huludao, Qinhuangdao, Tanggu and Longkou, were used to analyze the temporal and spatial characteristics of the storm surges in the Bohai Sea.

Clear seasonal variations exist in the storm surges in the Bohai Sea. The storm surges are more serious in the winter (from October to March) than those in the summer at all four tide gauges. The seasonal variability of the storm surges features few changes over the past few decades. A tide-surge interaction exists at all four tide gauges, and the distribution of the surge above the threshold values exhibited peaks during rising and falling tides. The decadal variations at the four tide gauges showed similar features. The frequency of the storm surge above the 99.9% level intensified after 2010. The four tide gauges showed some differences in the long-term trends of the storm surge count, duration and intensity. There were also some characteristics in common. The storm surge intensity showed a decreasing trend at QHD, HLD and TG, and the trends were significant at the 95% confidence level at QHD and TG.

The Arctic Oscillation (AO) and Siberian High (SH) greatly affect the cold-air outbreaks and extratropical cyclones over East Asia, which are the two main factors that generate storm surges in the Bohai Sea. It was found that a linear upward trend existed in the AO index from 1950 to 2016; meanwhile, the SH showed a decreasing trend. The storm surge intensity at LK and TG were significantly negatively correlated with the AO index, while significant positive correlations were found between the storm surge intensity and SH at HLD, QHD and TG. The results of the linear regression analysis strongly correspond to the storm surge intensity at the four tide gauges, especially at the long-term timescale. The changes in AO and SH can explain 19–48% of the changes in the storm surge intensity. The storm surge intensity from 1950 to 2016 was calculated using a linear regression model. The storm surge intensity showed a clear decreasing trend from 1950 to 2016 at all four tide gauges.

It should be noted that because of the data quality, in terms of both time period and spatial coverage, the results may be insufficient. It would be favorable if more data were made accessible for scientific analysis.

### Data availability

The data that support the findings of this study are available from the authors upon reasonable request.
